# Specifically Targeting Metacaspases of *Candida*: A New Therapeutic Opportunity

**DOI:** 10.3390/jof10020090

**Published:** 2024-01-23

**Authors:** Anne-Lise Bienvenu, Lionel Ballut, Stephane Picot

**Affiliations:** 1 Service Pharmacie, Groupement Hospitalier Nord, Hospices Civils de Lyon, 69004 Lyon, France; 2 Malaria Research Unit, University Lyon, UMR 5246 CNRS-INSA-CPE-University Lyon1, 69100 Villeurbanne, France; stephane.picot@univ-lyon1.fr; 3 Molecular Microbiology and Structural Biochemistry, UMR 5086, CNRS-Université de Lyon, 69367 Lyon, France; lionel.ballut@ibcp.fr; 4 Institute of Parasitology and Medical Mycology, Hôpital de la Croix-Rousse, Hospices Civils de Lyon, 69004 Lyon, France

**Keywords:** *Candida*, metacaspase, antifungal treatment, cell death

## Abstract

The World Health Organization (WHO) recently published a list of fungal priority pathogens, including *Candida albicans* and *C. auris*. The increased level of resistance of *Candida* is raising concern, considering the availability of only four classes of medicine. The WHO is seeking novel agent classes with different targets and mechanisms of action. Targeting *Candida* metacaspases to control intrinsic cell death could provide new therapeutic opportunities for invasive candidiasis. In this review, we provide the available evidence for *Candida* cell death, describe *Candida* metacaspases, and discuss the potential of *Candida* metacaspases to offer a new specific target. Targeting *Candida* cell death has good scientific rationale given that the fungicidal activity of many marketed antifungals is mediated, among others, by cell death triggering. But none of the available antifungals are specifically activating *Candida* metacaspases, making this target a new therapeutic opportunity for non-susceptible isolates. It is expected that antifungals based on the activation of fungi metacaspases will have a broad spectrum of action, as metacaspases have been described in many fungi, including filamentous fungi. Considering this original mechanism of action, it could be of great interest to combine these new antifungal candidates with existing antifungals. This approach would help to avoid the development of antifungal resistance, which is especially increasing in *Candida*.

## 1. Introduction

Invasive fungal diseases are responsible for over 300 million severe cases each year and 1.5 million deaths annually. According to the WHO, “fungal pathogens are a major threat to public health as they are becoming increasingly common and resistant to treatment with only four classes of antifungal medicines currently available, and few candidates in the clinical pipeline” [1]. Recently, the WHO published a list of fungal priority pathogens, including 19 fungi that represent the greatest threat to public health. The WHO fungal-priority-pathogens list serves to prioritize fungal pathogens, considering the unmet research-and-development needs and the perceived public-health importance [2]. Among the critical group of fungal pathogens are *Candida albicans* and *Candida auris* [2]. The yeast *Candida*, especially *C. albicans*, *Candida glabrata*, and *Candida parapsilosis*, is responsible for invasive infections of the blood, heart, central nervous system, eyes, bones, and internal organs associated with high mortality, especially in critically ill and immunocompromised patients. The overall mortality of invasive candidiasis ranges from 20% to 50%. Trends in *C. albicans* over the last 10 years are stable, but in-hospital infections caused by *C. albicans* are decreasing relative to more-resistant *Candida* species, including *C. auris* [3]. *C. auris* poses an emerging threat, as it is intrinsically resistant to most available antifungal medicines and some strains are pan-resistant [4]. *C. auris* is recognized as a highly transmissible healthcare pathogen and as a cause of hospital outbreaks. Patients with *C. auris* candidaemia had experienced longer length of stay in hospital than those with candidaemia caused by other *Candida* spp. [5]. 

The level of resistance of *Candida* species to azoles and echinocandins, both in clinical settings and in vitro, has increased in recent years [3]. There are three main ways in which *Candida* species may become resistant to azoles: the introduction of multidrug pumps in the fungal cell wall, allowing the cell to pump out the drug; alteration in the binding site of the enzyme lanosterol 14-α-sterol demethylase; and alteration of the fungal cell wall as a result of mutations [6]. For echinocandins, the resistance mechanism is explained by mutations in the FKS1 and FKS2 genes, which lead to changes in the target site [6]. The increased level of resistance of the *Candida* species responsible for infections in patients is raising concerns, considering the availability of only four classes of medicine. Azoles, echinocandins, pyrimidines, and polyenes are the four classes of systemic antifungal medicines used in clinical practice, and only a few others are under development [7]. The WHO is seeking novel agent classes with different targets and mechanisms of action. 

As proposed in the field of anti-cancer therapeutics, targeting *Candida* metacaspases, an ortholog of human caspase, to control intrinsic cell death, could provide new therapeutic opportunities for invasive candidiasis. Among new anti-cancer therapeutics, some approaches are based on apoptosis activation to reverse a common trait of malignantly transformed cells, which is the ability to evade apoptosis. Many clinically approved chemotherapeutic medications, including actinomycin-D, doxorubicin, topotecan, and bleomycin, exert potent antitumour effects by inducing regulated cell death modalities [8]. Apoptosis in humans involves a family of cysteine proteases known as caspases [9]. Caspases play a central role in the initiation and execution of apoptosis in metazoans. Activation of caspases leads to proteolytic events, including nuclear condensation, DNA fragmentation, and plasma-membrane blebbing, which contribute to dismantling the cell. Key regulators of caspase activity are the family of endogenous proteins named the inhibitor of apoptosis proteins (IAP). Therefore, several small-molecule mimetics have been developed in order to antagonise the IAP in cancer cells and restore sensitivity to apoptotic stimuli [10]. Due to their favourable safety profile but lower clinical efficacy than expected, small-molecule mimetics were proposed to be incorporated in combination therapies or as immuno-modulatory agents for the treatment of cancer [11]. Another anti-cancer approach to activate apoptosis is to specifically activate individual caspases through death receptors. Death receptors include the TNF-related apoptosis-inducing ligand (TRAIL) receptors (TRAIL-R1/DR4 and TRAIL-R2/DR5). TRAIL demonstrated a tumouricidal activity due to its ability to selectively kill cancer cells but not primary cells or tissue [12]. A number of TRAIL agonists, including agonistic antibodies against TRAIL-R1 and soluble recombinant forms of TRAIL, have been developed to clinically activate the TRAIL pathway for cancer therapies. To avoid resistance to these therapies, TRAIL agonists must be combined with other anti-cancer drugs, such as small-molecule mimetics [13]. Anti-cancer therapeutics based on the activation of apoptosis are then paving the way for new anti-*Candida* therapeutics based on the specific activation of *Candida* metacaspases.

The yeast metacaspase was described in *Saccharomyces cerevisiae*, whose genome encodes a metacaspase named Yca1 [14]. This metacaspase Yca1 was found to be involved in regulating cell death [15]. More recently, the first crystal structure of *C. glabrata* metacaspase was obtained, which allowed the identification of structural and molecular determinants of *C. glabrata* metacaspase activation by calcium [16]. Those findings are important prerequisites for the development of new antifungal strategies based on the activation of *Candida* metacaspases (CaMca1). This review aims at providing the available evidence for *Candida* cell death, then describing CaMca1, and, finally, discussing targeting CaMca1 as a new therapeutic opportunity.

## 2. Evidence for *Candida* Cell Death

The concept of programmed microbial death has been widely questioned in the last decade in part because authors did not use the same terminology to describe mechanistic and evolutionary cell death [17]. The mechanistic approach describing the complex biochemical machinery involved in cellular self-destruction is the most recognized approach in the field of mycology. For that purpose, assays used for the detection of mammalian cell death have been applied to yeast. The most common assays used to detect cell death in yeast, as well as cells from different origins, include fluorescence microscopy after Annexin V staining to detect the exposure of phosphatidylserine on the outer plasma-membrane leaflet, fluorescence microscopy or indirect tests for the TUNEL (terminal deoxynucleotidyl transferase dUTP nick-end labelling) assay to detect the nuclear DNA fragmentation, fluorescence microscopy after DAPI (4′,6-diamidino-2-phenylindole) staining to observe the chromatin condensation, microscopy quantification with antibodies specifically recognizing the active form of caspases or cleaved caspase substrates to detect the activation of caspase-like activity, and flow cytometry or fluorescence microscopy with specific markers to measure the mitochondrial membrane potential, the release of cytochrome c from mitochondria, or the production of reactive oxygen species (ROS) [18]. 

Yeasts, including *Saccharomyces* and *Candida*, are multifaceted fungi of high medical impact and considerable interest in science and biotechnology. The budding yeast *S. cerevisiae* is a fantastic model for cell biology and was one of the most used tools to study apoptosis [19]. Many of the pathways involved in accidental and regulated cell deaths were established from this cheap and easy-to-use model of eukaryotic cells [20]. Thus, it is now straightforward to use the accumulated knowledge on the fight against pathogenic yeasts in humans. Very useful guidelines were published to clarify the key concepts and the terminology of yeast cell death [19]. The nomenclature used in this study will follow these recommendations. Yeast cell death can be related to accidental cell death (ACD) following unfavourable microenvironmental conditions (high temperature, chemical or mechanical insults, or perturbation of high intensity) or regulated cell death (RCD) caused by apoptosis, autophagy, or programmed cell death (PCD). The death of the worst yeast cells may promote the growth of the fungal population in competitive conditions. The noteworthy example of old cells dying while releasing factors and nutrients favourable for the younger cells’ growth is a hallmark of the altruistic suicide [21]. But RCD in yeast may also be the result of competition for food, mating, or fighting against other populations [22]. RCD is executed by a genetically encoded dedicated machinery, which means that RCD can be modulated with drugs and pharmacological interventions [16]. Most of the morphological and biochemical changes observed in metazoans during RCD are similar to those observed in yeast, while the underlying mechanisms can be different depending on inducers. Some of the main changes described during RCD are externalization of phosphatidylserine; generation of ROS; DNA fragmentation; and loss of mitochondrial membrane potential, leading to the release of cytochrome c [23]. Hydrogen peroxide, acetic acid, formic acid, calcium stress, and UV irradiation are the most common chemicals or physical stimuli used to induce RCD in *S. cerevisiae* and *C. albicans* [22], but pharmacological stimuli have been also reported to induce RCD in *C. albicans*. Natural compounds, including amentoflavone [24], 1,2-benzopyrone [25], carvacrol [26], cinnamaldehyde [27], dill-seed essential oil [28], farnesol [29], hibicuslide C [30], isoquercitrin [31], limonene [32], naringin [33], nerol [34], oxyresveratrol [35], plagiochin E [36], propolis [37], purpurin [38], and resveratrol [39], as well as antimicrobial peptides, including arenicin-1 [40], coprisin [41], papiliocin [42], pleurocidin [43], psacotheasin [44], and scolopendin [45], were demonstrated to induce apoptosis in yeast, mostly in *C. albicans* and in a metacaspase-dependant manner. *C. albicans* yeast exposed to pleurocidin released intracellular ROS, especially hydroxyl radicals [43]. Arenicin-1 and papiliocin were also responsible for an increase in the production of ROS and cytotoxic hydroxyl radicals, leading to mitochondrial membrane depolarization and the release of activated metacaspases [40,42]. *C. albicans* cells treated with psacotheasin showed yeast apoptosis, including phosphatidylserine externalization, mitochondrial membrane depolarization, and increase of metacaspase activity [44]. Scolopendin led also to various apoptotic phenotypes, including ROS accumulation, phosphatidylserine exposure, chromatin condensation, and nuclear fragmentation [45]. Coprisin, which induced mitochondrial-membrane-potential dysfunction, cytochrome c release, and activation of metacaspases, accumulated in the nucleus of *C. albicans* cells [41]. 

Besides natural compounds and antimicrobial peptides, silver and gold nanoparticles exert their antifungal effect through apoptosis [46,47]. *C. albicans* cells exposed to silver nanoparticles showed increased ROS and hydroxyl radical production [46]. Gold nanoparticles interacted with *C. albicans* DNA, leading to increased nuclear condensation and DNA fragmentation, and with *C. albicans* mitochondria, leading to mitochondrial dysfunction [47]. However, gold nanoparticles induced ROS-independent apoptosis in *C. albicans*, considering that N-acetylcysteine, an ROS scavenger, did not influence the apoptotic pathway. 

Interestingly, some marketed antifungals, including amphotericin B [36], caspofungin [37], fluconazole [38], itraconazole [39], and micafungin [40], were also demonstrated to trigger yeast cell death. Most of these effects on apoptosis were reported in *C. albicans* yeast. Among the most frequent contributors to apoptosis in *C. albicans* after exposure to marketed antifungals are ROS release, mitochondrial dysfunction, cytochrome c release, and metacaspase activation.

## 3. Description of *Candida* Metacaspases

Metacaspases belong to the cluster-of-differentiation (CD) clan, a structural group of cysteine peptidases, and in the CD clan, to the C14 peptidase family (caspase family). Metacaspases are homologs of metazoan caspases as well as paracaspases. Considering that adequate classification and unified nomenclature of metacaspases and paracaspases is especially important to avoid frequent confusion of these proteases with caspases, a letter entitled “Classification and nomenclature of metacaspases and paracaspases: no more confusion with caspases” was published [48]. It provides a consensus opinion of researchers studying different aspects of caspases, metacaspases, and paracaspases in various organisms, ranging from microbes to plants and animals. It includes figures that compare domain composition and biochemical characteristics between caspases, metacaspases, and paracaspases. Three types of metacaspases and two types of paracaspases have been described [17,49]. All homologous groups of caspases have the histidine–cysteine (HC) catalytic dyad on the p20-like region. Type I metacaspase and paracaspase show an N-terminal pro-domain, while type II metacaspase and paracaspase, as well as type III metacaspases lack this prodomain. None of the metacaspases or paracaspases cleave after an aspartate residue: paracaspases are arginine-specific, whereas metacaspases can cleave after either arginine or lysine [48]. Apart from substrate specificity, active metacaspases are monomers, and their activation usually requires millimolar concentrations of calcium, whereas active caspases and paracaspases are calcium-independent dimers [48]. Metacaspases are found in protists, fungi, and plants but not in metazoan organisms, providing an interesting target specificity for future drug development [49]. Type I metacaspases from fungi share structural similarities with mammalian caspases, including a caspase-specific catalytic dyad of histidine and cysteine in the large subunit p20 and an N-terminal pro-domain, but they differ in their sequences, substrate specificity, and mechanisms of activation [50]. CaMca1 metacaspase is the single ortholog of the mammalian caspases in *C. albicans* and very similar to the Yca1 metacaspase of *S. cerevisiae* [51]. Metacaspases were also described in *C. parapsilosis* [52], *Candida tropicalis* [53], and *C. glabrata* [54].

**Figure 1:**
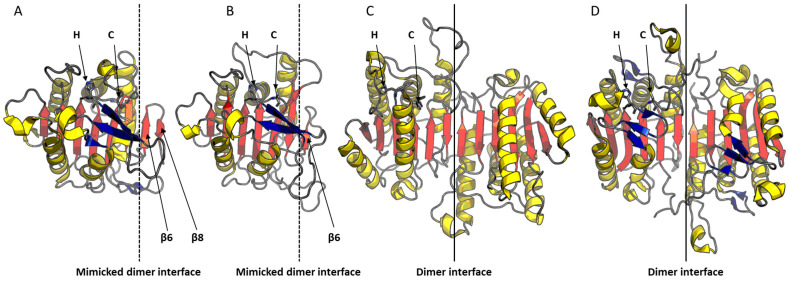
Comparison of the structures of *C. glabrata* metacaspase (CgMCA-I) and *C. albicans* metacaspase (CaMCA-I) with human caspase-6 and caspase-7. (**A**–**C**) Representation of the three-dimensional structure of the metacaspase CgMCA-I (PDB code: 7qp0) (A), the metacaspase CaMCA-I (AlphaFold) (**B**), caspase-6 (PDB code: 2wdp) (**C**), and caspase-7 (**D**) (PDB code: 3ibf). Loops are in gray, α-helices in yellow, β-strands of the central sheet in red, and additional β-strands in blue. The catalytic residues His (H) and Cys (C) are located at the C-terminal end of the β3 and β4 strands, respectively. The dimeric interfaces of caspase-6 and caspase-7 are represented by solid lines, and the mimicked dimeric interfaces of metacaspases are shown with dashed lines. In the AlphaFold representation of the *Candida albicans* metacaspase, the β8 strand is not modeled.

To date, only four three-dimensional structures were determined using X-ray crystallography. These include the type I metacaspases from the parasite *Trypanosoma brucei* (*Tb*MCA-Ib) [55] and the yeast *S. cerevisiae* (*Sc*MCA-I/Yca1) [56] described in 2012, followed by type II metacaspase-4 from the plant *Arabidopsis thaliana* (*At*MCA-IId) [57] and the type I metacaspase from the yeast *C. glabrata* (CgMCA-I) [16], respectively described in 2020 and 2022. These structures enabled the assessment of structural differences that distinguish them from effector caspases. Unlike mammalian caspases, which form dimers after maturation, it was observed that metacaspases remain in a monomeric form (Figure 1). The determination of these three-dimensional structures enabled also the assessment of similarities between metacaspases. In general, type I metacaspases exhibit the same structural organization as observed for *S. cerevisiae* and *C. glabrata* with a mixed beta sheet of eight strands of β (β1–β8), including six parallel and two anti-parallel strands (Figure 1). The catalytic residues histidine and cysteine are located on loops L3 and L4, respectively (Figure 1). Another interesting characteristic shared by the metacaspases is their calcium-dependent activity. The presence of calcium could contribute to both their maturation and increase their catalytic activity. We demonstrated that the binding of calcium to the metacaspase induced a conformational change, bringing the two catalytic residues closer together [16]. These shared characteristics of metacaspases among different organisms, combined with their significant divergence from mammalian caspases, make metacaspases the preferred therapeutic targets to induce RCD.

**Figure 2:**
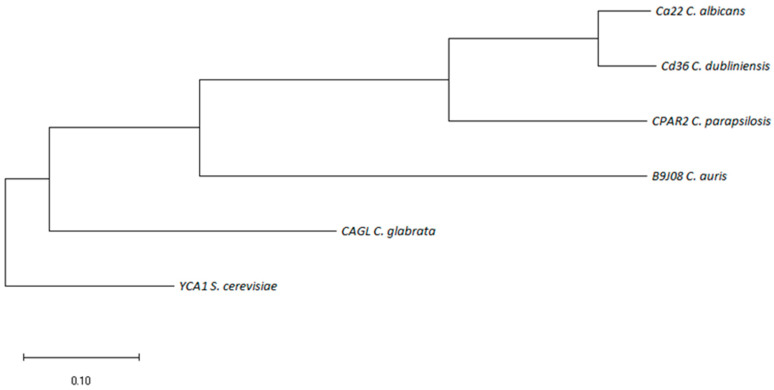
Evolutionary analysis by the maximum-likelihood method of the metacaspase orthologs of the *Candida* species rooted to YCA1 of *S. cerevisiae*. The evolutionary history was inferred by using the maximum-likelihood method and Tamura–Nei model [1]. The tree with the highest log likelihood (−7319.54) is shown. The tree is drawn to scale, with branch lengths measured in the number of substitutions per site. This analysis involved 6 nucleotide sequences. Codon positions included were 1st + 2nd + 3rd + Noncoding. There were a total of 1381 positions in the final dataset. Evolutionary analyses were conducted in MEGA (version X).

Metacaspases have more complex activities than only RCD since they have been also shown to maintain the proteasome and to be involved in virulence and pathogenicity. The balancing activity of yeast metacaspase between live or death of fungal cells has been extensively described and reviewed [20,22,58]. The role of CaMca1 in protein quality control through its interaction with protein aggregates confirmed also its non-death functions [20]. However, little is known about the machinery involved in *C. albicans* for RCD. Recently, database screening followed by overexpression analysis identified two putative pro-apoptosis factors: CaNma111, a homolog of the pro-apoptotic mammalian HtrA2/Omi, and CaYbh3, a homolog of BH3-only protein [59]. It is interesting to note that these factors were also shown to be involved in the hyperfilamentation phenotype and increased virulence, while the overlap with the pro-apoptotic activity is unclear [59]. A similar effect was previously observed with *C. albicans* metacaspase 1 (CaMca1) and EDC3p, a scaffold protein involved in mRNA decapping [51,60]. The number of DNA sequences of metacaspases from the *Candida* species is relatively limited in the *Candida* genome database (CGD) (http://www.candidagenome.org/ accessed on 2 December 2023). Putative metacaspase orthologs listed there are from *C. albicans* (MCA1/C3_05190C), *C. glabrata* (CAGL0I10945g/YCA1), *C. dubliniensis* (Cd36_85170), *C. parapsilosis* (CPAR2_405420), and *C. auris* (B9J08_001955). A phylogenetic tree of these sequences was prepared and rooted to YCA-1 from *S. cerevisiae* (Figure 2). This phylogenetic analysis suggests that there is a close proximity between the *Candida* species metacaspases that could be functionally replaced by orthologs. More specifically, *C. albicans* and *C. auris* metacaspases are probably not significantly distinct. The high identity rate between metacaspases of *Candida* indicates a strong structural conservation. The close proximity of *Candida* metacaspases combined with a strong structural conservation would facilitate the design of a pro-apoptotic compound able to induce the cell death of different *Candida*-related fungi. However, deciphering the highly regulated life-or-death decision in *C. albicans* needs to be elucidated before developing antifungal drugs with high target specificity.

## 4. Specifically Activating *Candida* Metacaspases: A New Therapeutic Opportunity?

There is an urgent need for new therapeutic options in the antifungal armamentarium given the emergence of resistant fungi to current antifungals. The recent emergence of multidrug-resistant *C. auris* and *C. glabrata* and acquiring invasive infections due to azole-resistant *C. parapsilosis*, *C. tropicalis*, and *Aspergillus* spp. in azole-naïve patients represent a serious health threat [61]. The hypothesis that an environmental source of resistant isolates was responsible for the emergence of azole resistance was supported by the identification of primary cases caused by azole-resistant isolates in patients who have never been treated with azoles [61]. Acquisition of environmental azole-resistant fungi, ranging from moulds to yeasts, then represents a great danger. Among the WHO priority areas for action is a focussed Research and Development investment in innovative antifungal agents (i.e., no cross-resistance to other antimicrobial classes, new chemical class, new target, and new mode of action) that are effective against priority pathogens [2]. Indeed, the four current antifungal agents indicated for the treatment of invasive fungal diseases are limited to three inhibition targets: ergosterol inhibitors (azoles and polyenes), 1,3-β-D-glucan synthase (GS) component FKS1 inhibitors (echinocandins and the newly approved ibrexafungerp), and pyrimidines interfering with RNA and DNA metabolism. Four promising antifungal agents, including fosmanogepix, olorofim, rezafungin, and opelconazole, are in late-phase clinical studies and will be available in the near future [7,62]. Future antifungals include first-in-class agents, new structures for an established target, and formulation modifications to marketed antifungals. Fosmanogepix is a first-in-class antifungal that inhibits the fungal Gwt1 enzyme that catalyses inositol acylation, which is an early step in the glycosylphosphatidylinositol (GPI)-anchor biosynthesis pathway [63]. Fosmanogepix obtained the orphan-drug designation in 2019. Orotomides are also a new class of antifungals that inhibits dihydroorotate dehydrogenase, a vital enzyme in fungal pyrimidine biosynthesis. Olorofim belongs to this new class [64]. Due to its potent antifungal activity on hard-to-treat fungi, including rare moulds, olorofim was recognized as an orphan drug by the U.S. Food and Drug Administration and by the European Medicines Agency Committee for Orphan Products. Regarding rezafungin and opelconazole, the first is a new structure for an established target and the second is a formulation modification to marketed azoles. Rezafungin is a novel echinocandin with an enhanced pharmacokinetic profile that allows rezafungin to be administered at extended intervals, such as once weekly [65]. Opelconazole is a triazole that was designed and optimized for inhalation via commonly available nebulizers [66].

As mentioned earlier, some marketed antifungals are known to trigger cell death. Caspofungin was demonstrated to exert its activity against *C. albicans* by causing necrosis and PCD associated with metacaspase activation: apoptosis was induced within the first hour of caspofungin exposure, and after 3 h of exposure, early apoptosis was observed in 20 to 25% of *C. albicans* cells [67]. Interestingly, *C. albicans* filamentous cells are more resistant to caspofungin and amphotericin B-induced PCD than blastospores: for that purpose, the viability of blastospores was compared to hyphal cells in media containing caspofungin or amphotericin B [68]. Filamentation appeared to protect yeast cells from caspofungin or amphotericin B-induced cell death, suggesting that the protective effects of filamentation may be a general phenomenon in *C. albicans*. The dose-dependent fungicidal activity of fluconazole in *C. albicans* is due to an apoptotic response confirmed by markers of apoptosis, including phosphatidylserine externalization and DNA damage [69]. Itraconazole was also demonstrated to induce apoptosis as a fungicidal mechanism in *C. albicans*, and intracellular ROS are a major contributor. Itraconazole also induced mitochondrial dysfunction, cytochrome c release, and metacaspase activation, which contribute to apoptosis [70]. Micafungin was shown to trigger caspase-dependent apoptosis in *C. albicans* and *C. parapsilosis* biofilms. Interestingly, this effect was similar in caspofungin-susceptible and caspofungin-non-susceptible isolates [52]. Given this, there is scientific evidence that targeting cell death contributes to fungicidal activity. 

As a proof of concept of this assertion, we tested the potential role of apoptosis in the antifungal activity of caspofungin [71]. Using a culture of *C. glabrata* (reference ATCC strain 7694), we demonstrated that caspofungin fungicidal activity was removed using Z-VAD-FMK, a well-known pan-caspase inhibitor including metacaspase. Z-VAD-FMK was used at a concentration of 40 µmol/L and caspofungin at a concentration of 0.5 mg/L, corresponding to twice the minimum inhibitory concentration (MIC) of *C. glabrata* (ATCC 7694). We used the EUCAST Definitive Document (E.Def) 7.4 method for the determination of broth-dilution minimum inhibitory concentrations for yeasts [72]. The test was performed in flat-bottom-well microdilution plates using RPMI 1640 supplemented with L-glutamine and glucose to a final concentration of 2%. As recommended, inoculum was obtained from a 24 h culture on nutritive-agar medium and prepared in sterile distilled water. The final inoculum was between 1 × 10^5^ CFU/mL. Each condition, i.e., caspofungin, caspofungin combined to Z-VAD-FMK, and control of growth, was performed in triplicates. Microdilution plates were incubated without agitation at 35 ± 2 °C in ambient air for 72 h. The microdilution plates were read after 24 h, 48 h, and 72 h of incubation with a microdilution plate reader at 530 nm. 

After 24 h of incubation, there was a significant growth (*p* = 0.007) of *C. glabrata* culture exposed to caspofungin and Z-VAD-FMK comparable to the control of growth, whereas *C. glabrata* culture exposed to caspofungin only was inhibited (unpublished data). In this experiment, we demonstrated that the pan-caspase inhibitor Z-VAD-FMK had the capability to significantly restore *C. glabrata* growth despite the presence of caspofungin at a concentration of twice the MIC. This experimental result corroborates the fact that the caspofungin fungicidal effect is mediated in part through metacaspase activation and that the antifungal effect of caspofungin can be reversed using a pan-caspase inhibitor. A similar result was obtained on *Candida* biofilm exposed to amphotericin B: using Z-VAD-FMK, authors showed that the viability of amphotericin B-treated biofilms increased up to 11.5-fold (*p* < 0.001) [73]. Thus, the caspase inhibitor Z-VAD-FMK is able to decrease the activity of amphotericin B against *Candida* biofilms. In that respect, it is possible to act on the apoptotic machinery of *Candida* in the aim of modulating *Candida* growth. Interestingly, it is expected that antifungals based on the activation of fungi metacaspases will have a broad spectrum of action, as metacaspases have been described in many fungi, including filamentous fungi like *Aspergillus* [74], *Fusarium* [75], and *Rhizopus* [76]. Two metacaspases, CasA and CasB, were described in *A. fumigatus* [74]. Using a metacaspase-deficient mutant, authors provided evidence that metacaspases are required for the optimal growth of *A. fumigatus* under conditions of endoplasmic-reticulum stress. In the *Fusarium graminearum* genome, two metacaspase genes (FGSG_12913 and FGSG_09204) were also identified [75]. 

Surfactin was able to activate gene expression of metacaspases and corresponding pathways to induce apoptosis in *F. graminearum* hyphae. Surfactin may then exert its antifungal activity against *F. graminearum* by activating apoptosis. In *Rhizopus oryzae*, iron starvation leads to physiological stress and metacaspase-dependent apoptosis [76]. Iron starvation was then identified as a critical component to induce metacaspase-dependent pathways that led to apoptosis. In filamentous fungi as previously discussed for *Candida* yeast, metacaspase-dependent apoptosis is a critical player in inducing fungicidal effects. 

**Figure 3:**
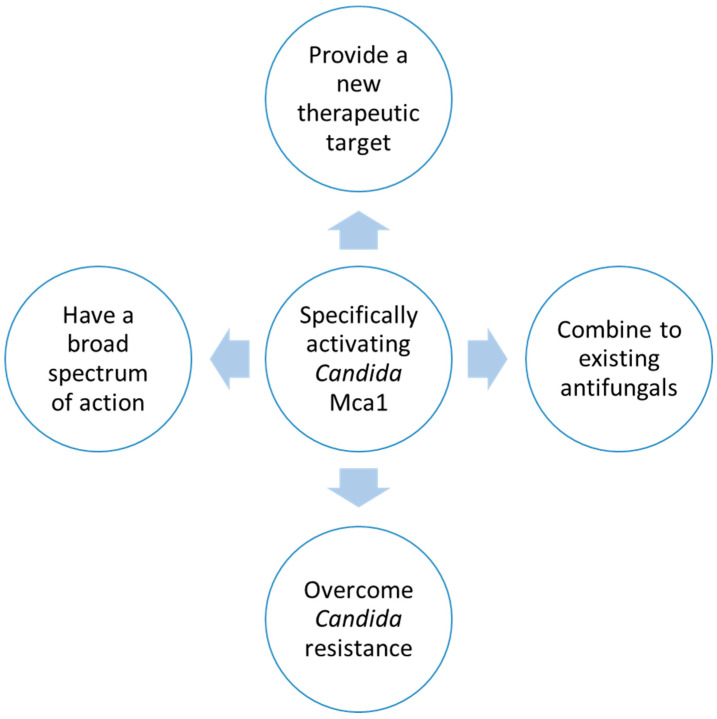
Rationale for specifically activating *Candida* metacaspases (Mca1).

Specifically activating metacaspases of *Candida* offers a new therapeutic opportunity for the treatment of infections caused by *Candida* or other fungi, providing the potential to develop broad-spectrum antifungals that could help to overcome resistance (Figure 3). 

## 5. Conclusions

Targeting *Candida* cell death has good scientific rationale given that the fungicidal activity of many marketed antifungals is mediated among others by triggering cell death [77]. However, none of the available antifungals specifically target *Candida* metacaspases, making this target a new therapeutic opportunity for non-susceptible isolates. Nevertheless, in-depth understanding of *Candida* RCD, especially the role played by the metacaspases in the life-or-death decision, is essential to develop new antifungals that specifically target *Candida* metacaspases. Whereas metacaspases are not present in mammalian cells, addressing the selectivity of the target (i.e., metacaspases of fungi) will also be a great challenge for the development of these new antifungal candidates. It is expected that antifungals based on the activation of fungi metacaspases will have a broad spectrum of action, as metacaspases have been described in many fungi, including filamentous fungi. Considering this original mechanism of action through targeting fungi metacaspases, it could be of great interest to combine these new antifungal candidates with existing antifungals. This approach would help prevent the development of antifungal resistance, which is especially increasing in *Candida* yeast. New highly resistant species are emerging, such as *C. auris*, and we must be ready to fight against an epidemic of resistant *Candida* in hospitals.
